# EVALUATION OF TENOTOMY IN THE HIP SUBLUXATION TREATMENT OF CHILDREN WITH ZIKA SYNDROME

**DOI:** 10.1590/1413-785220233101e256215

**Published:** 2023-04-17

**Authors:** Thiago Danilo Rodrigues de Almeida, Paulo Roberto Carvalho Carvalho, Brauner de Souza Cavalcanti, Gabriel Guerra Cordeiro, Caio César Barbosa Siqueira, Vinicius Gueiros Buenos Aires, EpitÁcio Leite Rolim

**Affiliations:** 1 Universidade Federal de Pernambuco, Getúlio Vargas Hospital, Recife, Brazil.

**Keywords:** Zika virus, Hip dislocation, Femoral head, Soft tissue release, Zika vírus, Luxação do quadril, Cabeça femoral, Liberação de Tecidos Moles

## Abstract

**Objectives:**

Evaluate the efficacy and effects of releasing the muscles of subluxated hips of patients with SCZ.

**Methods:**

This is a retrospective study with 29 patients with subluxation of the hip, corresponding to 55 hips operated in a public hospital in Recife, Brazil. Preoperative femoral head migration (PM) percentages were evaluated and compared with 6- and 12-month postoperative results.

**Results:**

Twenty-nine patients were eligible, representing 55 hips evaluated. 19 were female (65.5%), with a mean age of 31.45 months (ranging from 23 to 42 years). 19 patients were GMFCS level V (65.5%), 34.5% were level IV, and 20 of the 29 patients (69%) had no complications. The PM had an absolute reduction of 11.6% (GMFCS IV) and 13.31% (GMFCS V) in the first six months. After 12 months, there was a regression of MP of 7.14% (GMFCS V) and 11.25% (GMFCS IV) compared to preoperative values, with no significant statistical difference among MP values presented between 6 and 12 months after surgery.

**Conclusions:**

The surgery was effective in PM regression during the analyzed period and presented a low complication rate. ***Level of Evidence III; Comparative retrospective study.***

## RESUMO

**Objetivos:**

Avaliar a eficácia e os efeitos da liberação das musculaturas de quadris subluxados de pacientes com SCZ.

**Métodos:**

Trata-se de um estudo do tipo retrospectivo com 29 pacientes CZS com subluxação do quadril, correspondendo a 55 quadris operados em um hospital público de Recife, Brasil. Foram avaliados os percentuais migratórios da cabeça femoral (PM) no pré-operatório e comparados com os resultados pós-operatórios de 6 e 12 meses.

**Resultados:**

Foram elegíveis 29 pacientes, representando 55 quadris avaliados. 19 eram do sexo feminino (65,5%), com média de idade de 31,45 meses (variando de 23 a 42 anos). 19 pacientes eram GMFCS nível V (65,5%), 34,5% eram nível IV e 20 dos 29 pacientes (69%) não apresentaram complicações. O PM teve redução absoluta de 11,6% (GMFCS IV) e de 13,31% (GMFCS V), nos primeiros seis meses. Após 12 meses, houve regressão do PM de 7,14% (GMFCS V) e de 11,25% (GMFCS IV) em relação aos valores pré-operatórios, sem diferença estatística entre os valores do PM apresentados entre 6 e 12 meses da cirurgia.

**Conclusões:**

A cirurgia foi eficaz em regredir o PM no período analisado e apresentou uma baixa taxa de complicações. ***Nível de Evidência III; Estudo retrospectivo comparativo.***

## INTRODUCTION

Congenital Zika syndrome (CZS) is a Brazilian public health problem^1^, it presents a distinct pattern of birth defects and disabilities resulting from intrauterine Zika Virus (ZIKV) ^2^ infection, may have orthopedic^3^ and neurological repercussions^3,4^ which include important spastic hypertonia^5,6^ with early hip dislocation, besides other systemic changes^7-9^. The motor impaiment in these children is mostly grave. According to Melo^6^, the gross motor function classification system (GMFCS)^10^ can be used in CZS, with 81% of children presenting with level V^6^. As in cerebral palsy (CP), they are prone to hip dislocation due to spasticity. This should be treated or avoided, regardless of the gait prognosis, as it has a negative impact on patients’ quality of life^11-13^.

In subluxated hips, soft tissue release is a surgical procedure used in patients with CP, with proven effectiveness in preventing spastic dislocation of the hips^14-17^, especially in young patients, under 4 years old. In many cases bone reconstructive surgery may be necessary^14,18^, but it is usually reserved for patients over 4 years old^15,19^.

In our study all our patients were younger than 4 years old, therefore unfit to undergo osseous reconstructive surgery.

However, there are no studies evaluating the effectiveness of soft tissue release in hip subluxation of CZS patients. Patients with CZS are currently being treated, in some Brazilian medical centers, according to the guidelines for CP patients.

In a pioneering way, has been evaluated the efficacy of soft tissue release from the hip in CZS patients with subluxation in delaying natural progression of spastic hip dislocation. These patients were assessed after a follow-up period up to 12 months. This is a preliminary study aimed to evaluate the efficacy of tenotomies in the hips of CZS patients under 4 years old, who presented hip subluxation, using the migration percentage of the femoral head (MP) on the pelvic radiograph, according to Reimers index method^20^, as a measure to assess the outcome of subluxation within a year after surgery. New studies will be conducted to assess these long-term effects.

## MATERIAL AND METHODS

The study population comprised a consecutive series of forty-two children with CZS, from 23 to 42 months old, who underwent surgery between January 2017 and December 2018, in a public hospital in Recife, Pernambuco state, Brazil, being submitted to open tenotomy to release the hip adductors and of the iliopsoas, for treating spastic subluxation of the hip. Postoperatively, all were included in motor rehabilitation programs after a period of 30 to 60 days of use a hip abduction orthosis. The board of the Research Ethics Committee from the Federal University of Pernambuco approved this study, waiving the use of informed consent (CAAE: 87564218.2.0000.5208). A retrospective review of the results in this group was performed in two different periods, defined at six and twelve months after surgery. Patients in this study had to meet the following inclusion criteria: have clinical and laboratory confirmation of CZS; present at least one hip at risk of dislocation prior to surgery (defined as an MP of ≥ 30% or hip abduction of 30° or less); have medical records correctly filled in, with clinical and radiographic data (pre and postoperative); and have not undergone previous hip surgeries.

The study was retrospective, observational, and longitudinal. Preoperative, six-month and twelve-month postoperative radiographs were reviewed to determine MP. Patients’ medical records were reviewed to determine GMFCS motor level record, outpatient status, age at surgery, hip abduction, and surgical history of repeated soft tissue release or bone surgery on the hip. Data was collected between May 15, 2019, and August 30, 2020. Forty-two children were selected for the study. All 42 patients had CZS with subluxation of at least one hip and surgical indication signed by a pediatric orthopedist. Of these, 13 children were excluded: 5 for partial or total loss of data from their medical records; 2 for not having the laboratory confirmation of CZS's diagnosis in their medical records; and 6 for not having radiographic data stored in their medical records, or by presenting inadequate radiographs for radiographic index evaluation. Ultimately 29 patients met the inclusion criteria. All patients were unable to walk, 19 patients (65.5%) were female, and the others were male. The patients were, on average, 31.45 months-old at the time of surgery (ranging from 23 to 42 months). The majority had GMFCS level V (65.5%).

All patients underwent surgery with the same team of orthopedists, following American Academy of Cerebral Palsy and Developmental Medicine (AACPDM)^21^ recommendations to classify the patient's risk of having a hip dislocated, and were guided by the existing criteria for CP used by Presedo^15^ and other authors^14,16,17,20,22^ to indicate the surgeries. Surgical indications were based on the patient's age, degree of hip abduction in flexion, and percentage of hip migration (MP ≥ 30% or hip abduction of 30° or less)^21^. The surgical procedure comprised tenotomy of the long adductor, of the short adductor, iliopsoas and complete myotomy of the gracilis in all patients.

**Figure 1:**
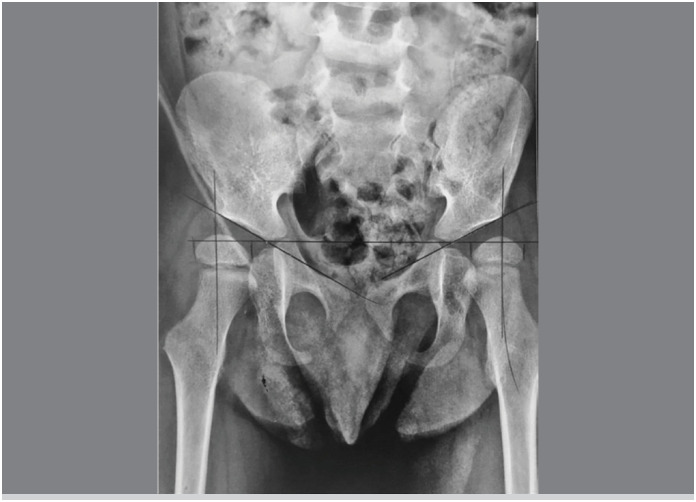
Anteroposterior radiography of the pelvis with migration percentages of the femoral head measurement.

This procedure was performed on both hips in twenty-six of the twenty-nine patients. Asymmetric procedures, defined as operations in which the soft-tissue release was performed unilaterally, were carried out in three patients. In total, fifty-five hips were submitted to this method. Hip migration percentage (MP) was used to determine the degree of hip subluxation radiographically, according to parameters described by Reimers^20^. To determine the MP, anteroposterior (AP) radiographs of the pelvis were used, taken up to 30 days before surgery and in the postoperative periods of 6 and 12 months, performed with the patient in supine position, femurs in neutral abduction-adduction in relation to the pelvis, and the patella facing forward. (Figure 1)

The same investigator analyzed all the patients’ radiographic indexes. Each radiograph received a random identification code that did not allow the evaluator, during the analysis, to identify the patient to which they belonged, and made no reference to the period when they were taken (preoperative, 6 months or 12 months postoperative).

### Statistical Analysis

Patients’ clinical data, such as age at the time of surgery and complication rate, were analyzed descriptively, using absolute frequencies and percentages for categorical variables, and measures such as mean and standard deviation (mean ± SD) for numeric variables. In MP evaluation, in pre and postoperative periods, a category comparison was made in relation to the numerical variables (scale scores) and the following tests were used: Student's t-test or Mann-Whitney for two categories, and F (ANOVA) or Kruskal -Wallis for more than two categories. When comparing pairs of assessments on numerical variables, paired Student's t-test or paired Wilcoxon tests were used. The difference between values obtained in the scale means and reference values was verified using a one sample t-test. Student's t-tests were used for equal variances. Student's t-test for unequal variances, paired Student's t-test, F (ANOVA) and Pearson's correlation occurred in situations where the data (or the difference between evaluations) presented a normal distribution. Mann-Whitney, paired Wilcoxon and Kruskal-Wallis tests were used due to the absence of normality, sample size inferior to 6 cases or ordinal scale. The verification of normality was performed by the Shapiro-Wilk test and equality of variances by the Levene F test. The level of significance was set at p ≤ 0.05.

## RESULTS

Twenty-nine CZS patients with spastic hip subluxation underwent soft tissue release procedures, in a total of 55 hips. Patients were, on average, 31.45 months old at the time of the surgery (standard deviation of 5.34 months, median of 30.00 and range from 23 to 42 months). Table 1 shows that 51.7% were 24 to 30 months old, 20.7% were 31 to 36 months old, and 27.6% were 37 to 42 months old. Regarding the GMFCS classification, 65.5% presented level V and 34.5% were level IV, no patient had GMFCS I, II or III. 69% patients did not report complications, but 31.0% of them had some complication, the most prevalent of which was irritability (13.8%), followed by genital edema (10.3%) and contracture of hips in abduction (10.3%). Seizures and fever had reported frequencies of 6.9%, pain was reported in 3.4%. There was no record of suture dehiscence, postoperative infection, vascular or nerve injury or death resulting from surgery. (Table 1)

**Table 1:** Demographic profile of the analyzed sample.

Variable.	n	%
Total	29	100.0
**Age group (months)**		
24 to 30	15	51.7
31 to 36	6	20.7
30 to 42	8	27.6
**Sex**		
Male	10	34.5
Female	19	65.5
**GMFCS**		
IV	10	34.5
V	19	65.5
**Occurrence of complications**		
Yes	9	31.0
No	20	69.0
**Complications**		
None	20	69.0
Irritability	4	13.8
Edema	3	10.3
Abduction contracture	3	10.3
Convulsion	2	6.9
Fever	2	6.9
Pain	1	3.4

All children had at least one hip with subluxation, defined as a migration percentage ≥30%. Of the 29 patients who underwent surgery, 26 were operated on both hips and 3 were operated unilaterally, totaling 55 evaluated hips. Table 2 shows the MP (in %), according to the GMFCS, and the comparative evaluations between pre (operative) and 6 months (post-operative), pre and 12 months, and 6 months to 12 months. (Table 2)

**Table 2:** Comparative statistics on MP according to GMFCS and operative period.

	GMFCS		
Variable	IV	V	p-value
	Mean ± SD (median)	Mean ± SD (median)	
**MP (in%)**			
**Pre × 6 months**			
Pre (n = 39)	37.80 ± 11.25 (37.00)	53.00 ± 17.76 (50.00)	p(1) = 0.016 *
6 months (n = 39)	26.20 ± 11.92 (30.00)	39.69 ± 11.25 (40.00)	p(1) = 0.003 *
p-value	p(4) = 0.013 *	p(5) <0.001 *	
Absolute difference	11.60 ± 8.22 (9.00)	13.31 ± 16.43 (8.00)	p(2) = 0.489
Difference %	32.41 ± 21.75 (26.72)	21.27 ± 19.75 (21.05)	p(2) = 0.126
**Pre × 12 months**			
Pre (n = 41)	48.42 ± 14.52 (46.00)	48.59 ± 14.17 (47.00)	p(1) = 0.973
12 months (n = 41)	37.17 ± 15.13 (31.50)	41.45 ± 18.41 (39.00)	p(1) = 0.481
p-value	p(4) = 0.015 *	p(4) = 0.052	
Absolute difference	11.25 ± 13.62 (9.00)	7.14 ± 18.94 (4.00)	p(1) = 0.500
Difference %	20.97 ± 25.17 (20.53)	11.23 ± 37.23 (10.00)	p(1) = 0.413
**6 months × 12 months**			
6 months (n = 25)	35.00 ± 7.75 (33.00)	41.48 ± 12.36 (44.00)	p(2) = 0.413
12 months (n = 25)	31.25 ± 7.46 (29.00)	39.90 ± 15.39 (38.00)	p(2) = 0.265
p-value	p(5) = 0.125	p(5) = 0.807	
Absolute difference	3.75 ± 0.50 (4.00)	1.57 ± 11.00 (0.00)	p(2) = 0.298
Difference %	10.92 ± 1.65 (11.24)	1.78 ± 30.13 (0.00)	p(2) = 0.207

(*) Significant difference at the level of 5.0%.

(1) By the Student's t-test with equal variances.

(2) By the Mann-Whitney test.

(3) By the Student's t-test with unequal variances.

(4) By the paired Student's t-test.

(5) By the paired Wilcoxon test.

According to results in Table 2, it can be noted that the only significant differences between the categories of GMFCS IV and V occurred in the Reimers index in pre and 6 month evaluations and, in these variables, the averages were found to be correspondingly higher among GMFCS V patients than in level IV patients (53.00 × 37.80 in the preoperative period and 39.69 × 26.20 in the 6 month evaluation). The averages were correspondingly higher in the pre rather than the 6 month assessment and in the pre rather than the 12 month assessment, with significant differences between pre and 6 month assessment in group IV and pre and 6 month assessment in group V. There were no significant differences (p> 0.05) between 6 and 12 months.

## DISCUSSION

The analysis of 29 CZS patients in this report revealed an early tendency to hip dislocation in CZS (average age of 31,45 months) may be related to the grave profile of spasticity in patients with the syndrome (usually GMFCS V). Despite a reportedly high failure rate in isolated soft tissue releases^22^, the patient population had a very young age profile, therefore the utilization of soft tissue releases, even in patients with a high MP (> 50%), was preferrable to reconstructive bone surgery, which may be performed with less surgical risk posteriorly if indicated. In our sample, all patients presented either GMFCS level V (65.5%) or level IV (34.5%). This profile of more grave involvement in CZS was also observed by Melo et al., who described in their sample a predominance of motor level V (GMFCS) in 81% of the children^6^. Another factor that could have influenced the early hip displacement in these patients may be the fact that, in the sample, no child was able to walk^12^.

The main reported surgical complication was the presence (or worsening) of irritability in the postoperative period (13.8%). The presence of irritability, defined by increased crying, is reported in CZS patients^5,6^, even those who have not undergone surgical procedures.

Evaluating the MP before and after 6 months of surgery, it was observed that the procedure was effective in regressing the MP, similarly in both level IV and level V groups. In patients with GMFCS IV (mean preoperative MP of 37, 8%), the MP decreased to 26.2%. In GMFCS V patients, the average MP was 53% and decreased to 39.69%.

According to Presedo et al., who defined in their study as satisfactory results those which, after the soft tissue release, had MP ≤39% and unsatisfactory those with MP ≥40%, the result presented in patients with CZS after 6 months is considered reasonably satisfactory (migration from 25% to 39%)^15^.

In the GMFCS V group, the MP regressed from 48.59% to 41.45%, 12 months after surgery. The MP 41.45% (GMFCS V) and 37.17% (GMFCS IV) is satisfactory if analyzed by the most current criteria used by Terjesen et al.^16^, who considers the surgery to be satisfactory (“successful”) when the MP in the last follow-up was <50%. Myongsu Ha et al.^24^ had a greater regression in the MP, in which the surgery reduced the MP from 62% to 37,9% on average.

Shore et al^22^ defined as a good result for soft tissue surgery the absence of revision surgery and a MP <50% at the last follow-up. 27% in our group (12 of the 44 hips evaluated in 12 months) had an unsatisfactory MP (50% or more), but the group had an average MP of 41.45% (GMFCS V) and 37.17% (GMFCS IV) in the last follow-up (12 months). Other researchers had not acceptable results: in 48% patients from Nikolaos Kiapekos et al. (GMFCS IV and IV) ^25^, 40% from Presedo et al.^15^, 58% from Turker and Lee^23^, and 75% of Shore^22^ et al. patients, they did not have satisfactory results by the same criteria. We had no cases of revision surgery.

Examining the MP before and 12 months after surgery, it is observed in the GMFCS IV group that the average MP after 12 months was 37.17%, showing an absolute reduction of 11.25, similar to the absolute decrease presented in the 6 month postoperative period (11.60), indicating that, in relation to the preoperative period, there was a reduction in MP, but comparing the 6 and 12 month postoperative periods, there was no statistical difference in the hip MP of these patients (p = 0.125 and absolute difference of 3.75), indicating a stabilization in the hip MP between 6 and 12 months after the surgery. Similarly to level IV, the level V group also presented a tendency towards stabilization in the MP between 6 and 12 months after surgery, with an absolute difference of only 1.57 and a percentage of 1.78%, showing no statistical difference between values measured at twelve and six months postoperative (p = 0.807).

The present study contributes to understanding the soft tissue surgery in the treatment applied in hip dislocation in CZS, contributing to assist orthopedists in the treatment of these patients. In our population, with a rare syndrome, we have had as limitation a small sample size. To expand this sample, we evaluated individualized results by hip. Another limiting factor was a short follow-up, as future perspectives, we intend to do new studies with longer follow-ups to determine if the MP regression persists more than 12 months after surgery and conduct new studies evaluating the results of reconstructive surgeries in dislocated hips of the SCZ.

## CONCLUSION

In conclusion, not all patients analyzed was a satisfactory final MP, but the surgery was effective in causing regression in MP, both in patients with GMFCS IV and V, with a significantly greater reduction in the first 6 months after the procedure, showing better results in level IV patients during this period. There was a tendency to stabilize the average MP obtained between 6 and 12 months after surgery. The surgery presented a low rate of complications in SCZ patients.
